# Management of fragile X-associated tremor ataxia syndrome: a review for clinicians

**DOI:** 10.3389/fneur.2026.1870779

**Published:** 2026-10-07

**Authors:** Deborah A. Hall, David Andrés González, Jessica Klusek, Peter K. Todd, Joan A. O’Keefe, Nell Maltman, Federica Alice Maria Montanaro, Dragana Protic, Laura Scorr, Rabia Malik, Pierpaolo Turcano, Matthew S. Meyers, Andreea Seritan

**Affiliations:** 1 Department of Neurological Sciences, Rush University Medical Center, Chicago, IL, United States; 2 Department of Communication Sciences and Disorders, University of South Carolina, Columbia, SC, United States; 3 Department of Neurology, University of Michigan, Ann Arbor, MI, United States; 4 Veterans Administration Ann Arbor Healthcare System, Ann Arbor, MI, United States; 5 Department of Speech, Language and Hearing Sciences, University of Arizona, Tucson, AZ, United States; 6 Department of Translational Biomedicine and Neuroscience (DiBraiN), University of Bari Aldo Moro, Bari, Italy; 7 Department of Pharmacology, Clinical Pharmacology and Toxicology, Faculty of Medicine, University of Belgrade, Belgrade, Serbia; 8 Department of Neurology, Emory University School of Medicine, Atlanta, GA, United States; 9 Department of Psychiatry and Behavioral Sciences, University of California, San Francisco, San Francisco, CA, United States; 10 UCSF Weill Institute for Neurosciences, San Francisco, CA, United States

**Keywords:** cerebellar ataxia, FXTAS (Fragile X-associated tremor/ataxia syndrome), motor dysfunction, rehabilitation, treatment

## Abstract

Fragile X-associated tremor ataxia syndrome (FXTAS) is a multi-faceted and progressive neurodegenerative disorder that manifests clinically with action tremor, gait ataxia, parkinsonism, peripheral neuropathy, and cognitive and psychiatric features. The disease is highly heterogeneous and there are no FDA approved medications for FXTAS as a specific indication. As such, treatment is symptomatic and must be tailored to the specific needs of a given patient. This review provides expert opinion-based treatment recommendations for the core and ancillary clinical features of FXTAS. Movement disorders, such as tremor, ataxia and parkinsonism may respond to symptomatic medications and therapies, but caution is required when considering surgical interventions such as deep brain stimulation. Cognitive decline can be responsive to medications developed for other cognitive disorders, but these medications work best when combined with non-medication based approaches and all patients should be screened for other reversible causes of memory decline. Psychiatric symptoms, including anxiety, depression, and psychosis, can be treated with psychotherapy and medications; but caution is required to avoid exacerbating clinical and subclinical movement disorder features with psychotropic medications. Rehabilitative therapies, such as speech therapy, occupational, and physical therapy, often enhance patient function and quality of life. Exercise and good nutrition are recommendations that are common to other cerebellar ataxias and adult neurodegenerative disorders and may have a positive impact on general health. A multi-disciplinary approach is recommended to address all components of FXTAS.

## 1 Introduction

Fragile X-associated tremor ataxia syndrome (FXTAS) is a neurodegenerative disorder that is characterized by movement disorders, cognitive decline, and other neurological signs (1). It is typically caused by a 55–199 CGG repeat expansion in the *fragile X messenger ribonucleoprotein 1 (FMR1)* gene. Affected individuals have onset usually over the age of 55 and there are characteristic imaging findings and neuropathology. Although onset of the disease was originally defined as onset of tremor, it is now known that up to 25% of FXTAS patients do not have tremor and may present with a variety of movement, cognitive or psychiatric signs (2, 3).

The evolution of neurological signs and symptoms is also highly variable, but progression occurs over decades with more severe disability typically occurring 15–20 years after symptom onset (4). Imaging findings including white matter lesions on T2-weighted MRI in the middle cerebellar peduncle (MCP sign), brainstem, or splenium of the corpus callosum are common (5). On neuropathology, eosinophilic intranuclear inclusions are present, in addition to widespread cortical and cerebellar atrophy, glial dysregulation (6), astrocyte degeneration (7), and cerebral microbleeds (8). The CGG repeat expansion is thought to elicit disease through gain-of-function mechanisms involving RNA mediated toxicity and repeat-associated non-AUG initiated (RAN) translation of polyglycine containing proteins (9, 10). Clinical penetrance and symptom severity correlate with both larger repeat size and sex, with higher penetrance in men (11). Diagnostic criteria for FXTAS requires a combination of neurological features and imaging findings, in the setting of a *FMR1* repeat expansion (Table 1) (5).

**Table 1:** Diagnostic criteria for Fragile X-associated tremor ataxia syndrome.

Diagnostic categories	
Definite:	Presence of one major radiological sign plus one major clinical symptom
Probable:	Presence of either one major radiological sign plus one minor clinical symptom or has the two major clinical symptoms
Possible:	Presence of one minor radiological sign plus one major clinical symptom
Molecular criteria	
Required	*FMR1* premutation or gray zone alleles (CGG 41–199)
Clinical criteria	
Major	Intention tremor
Major	Cerebellar gait ataxia
Minor	Parkinsonism
Minor	Neuropathy
Minor	≥Moderate to severe short-term memory deficiency
Minor	Executive function deficit
Radiological	
Major	MRI white matter lesions in middle cerebellar peduncle (MCP) and or brain stem
Minor	MRI white matter lesions in the splenium of the corpus callosum
Minor	MRI white matter lesions in cerebral white matter
Minor	≥ Moderate to severe generalized atrophy
Neuropathology	
Major	Ubiquitin-positive intranuclear inclusions

The prevalence of FXTAS varies according to sex and age of the carrier, due to the gene being located on the X chromosome. Overall, it may affect close to 150,000 individuals in the US based on the prevalence of the *FMR1* premutation in the population: from 1/148–1/209 women and 1/290–1/468 men (12). FXTAS is a progressive disorder with gradual decline to worsening mobility and cognitive deficits, leading to infection and death with 15–20 years of symptom onset.

There are few clinical trials of medications in FXTAS. This creates an unmet clinical need to provide guidance to treating physicians on how to manage the variety of symptoms in the disorder. In 2025, the National Fragile X Foundation asked clinicians and researchers at the International Fragile X Premutation meeting to update guidance to clinicians for posting on their FXTAS website as the current guideline articles on the site are from almost 20 years old. This paper was written in response to that request and will (1) replace the current papers on the website and (2) serve as the basis for a patient facing paper with treatment recommendations also from the National Fragile X Foundation. All attendees at the International Fragile X Premutation meeting were queried to contribute and they were invited to participate based on their experience, availability to contribute, and willingness.

In general, FXTAS is heterogeneous and individual patients should be treated tailored to their own set of symptoms. This is consistent with prior studies, that show a wide variety of medical classes and therapeutic options that may be beneficial for FXTAS patients (13). In this paper, a short review of clinical features of FXTAS is followed by treatment recommendations. Ongoing studies to develop clinical trial readiness in FXTAS will ideally lead to a wider array of options for patients in the future.

## 2 Methods

The narrative expert review was conducted by experts in the following areas: neurology, psychiatry, neuropsychology, neuromuscular medicine, physical therapy, speech therapy, and pharmacology. The PubMed (National Library of Medicine) database was searched with keywords (FXTAS, Fragile X Tremor Ataxia Syndrome, and Treatment). Results were filtered for articles related to humans published within the last 10 years. This search produced 66 results. These results were reviewed to identify publications with information pertaining to the treatment of FXTAS signs and symptoms. Where there was no data in FXTAS or low level data, additional sources on standard management of signs and symptoms, which are not specific to FXTAS, were also referenced in the discussion for context. Final treatment recommendations were based on published with level of evidence based on American Academy of Neurology recommendations included (see Figures 1–3).

**Figure 1:**
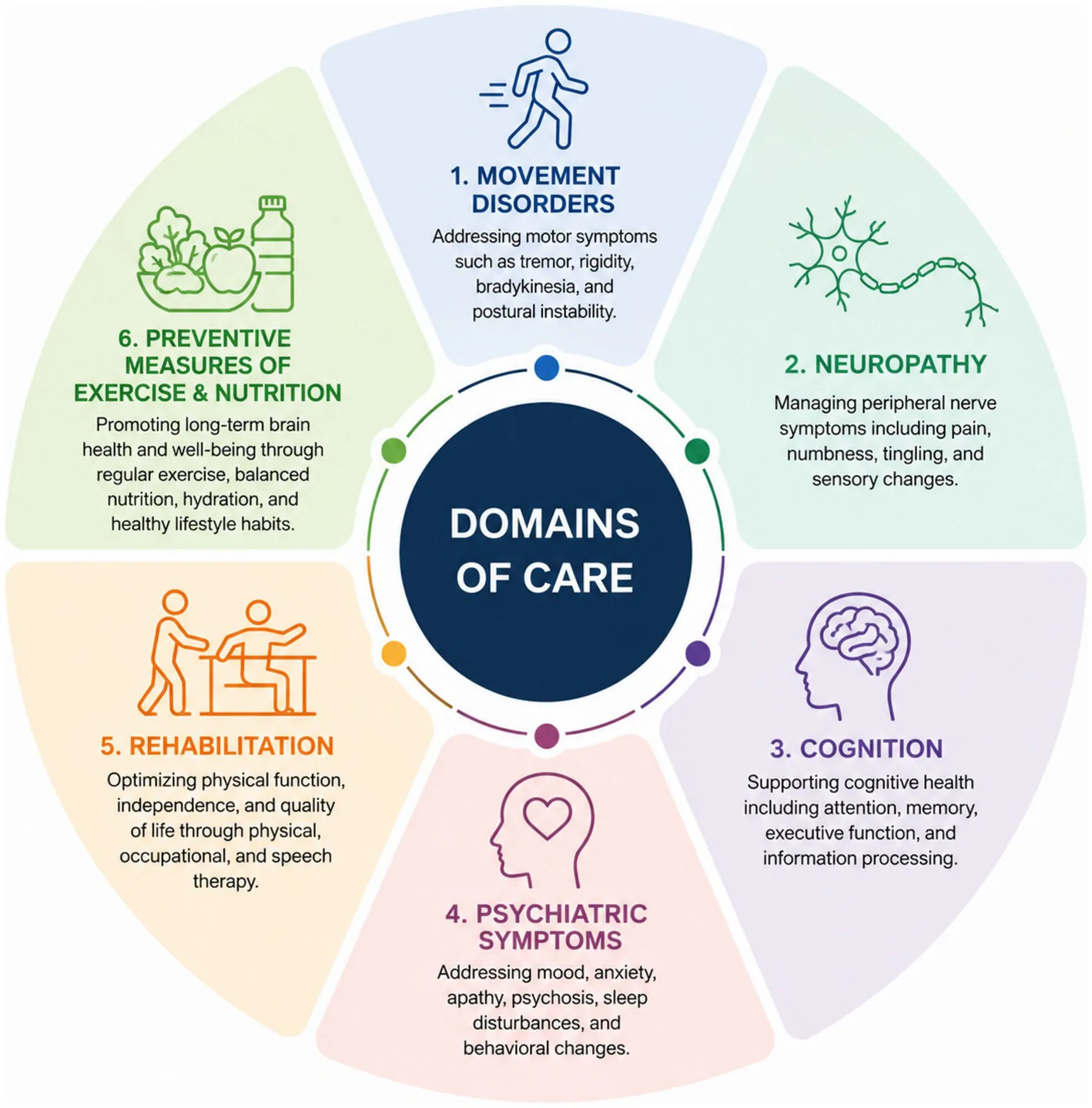
Domains of care for Fragile X-associated tremor ataxia syndrome.

**Figure 2:**
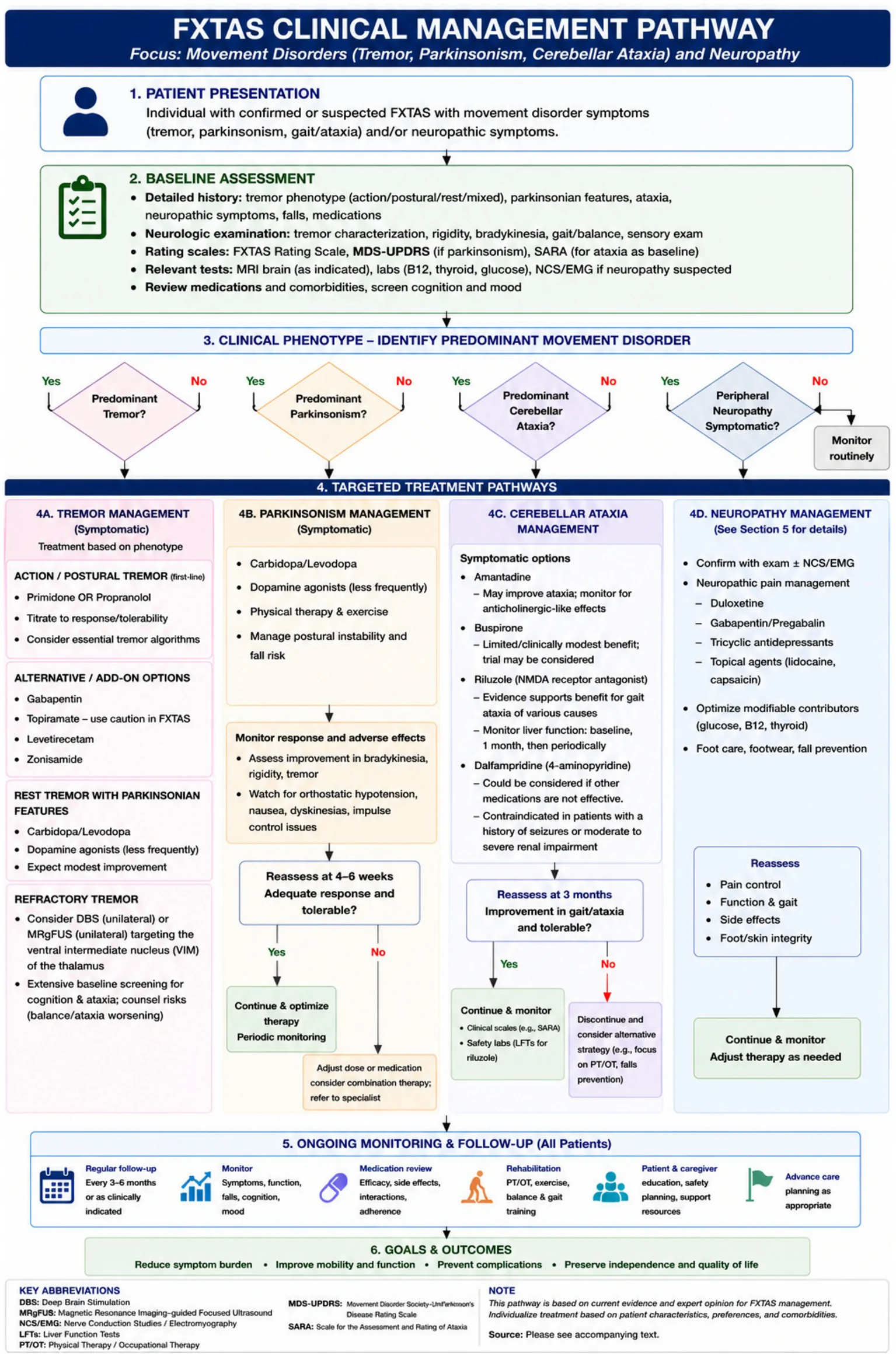
Clinical management pathway for movement disorders in Fragile X associated tremor ataxia syndrome (FXTAS).

**Figure 3:**
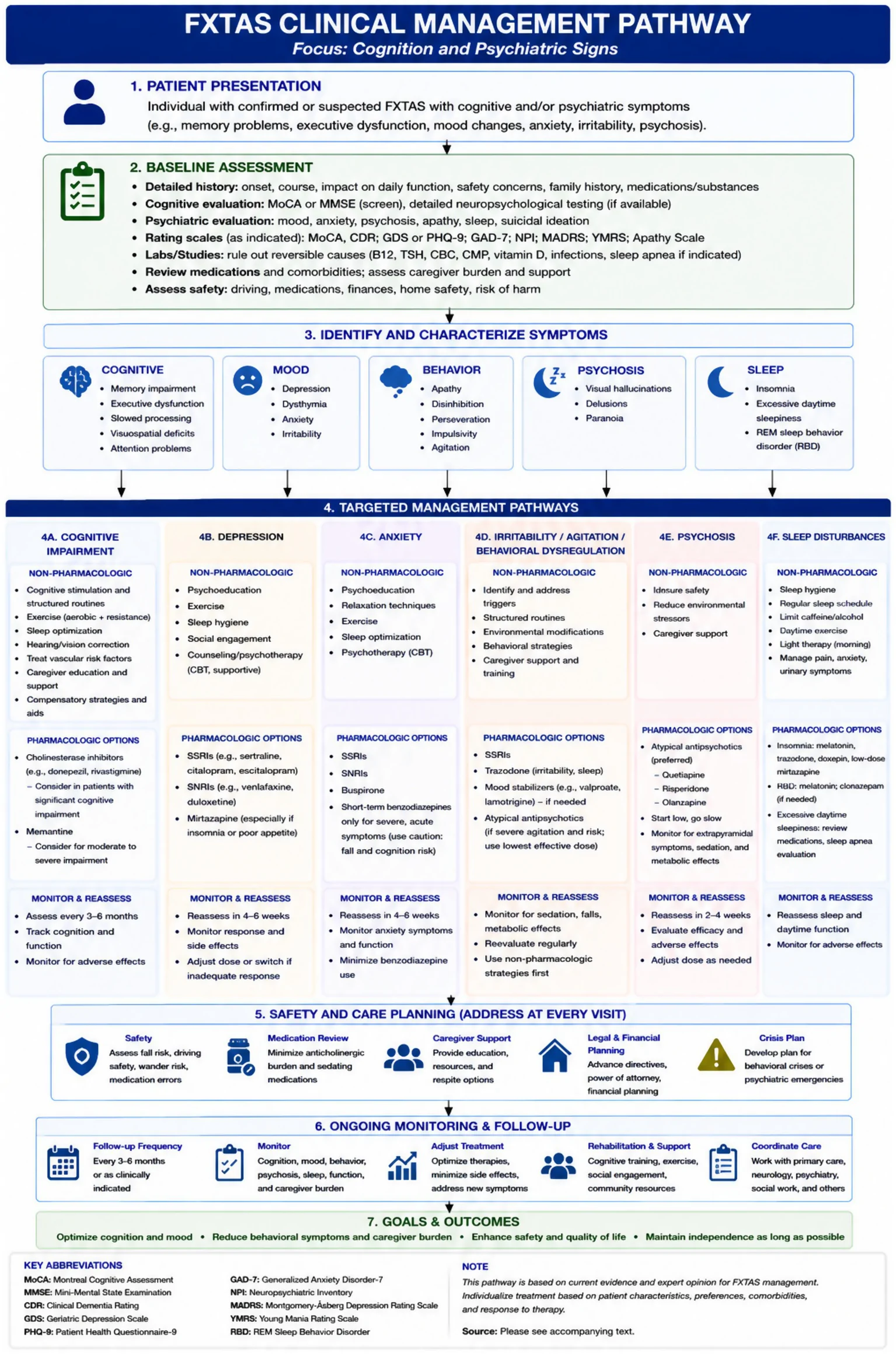
Clinical management pathway for neuropsychiatric signs in Fragile X associated tremor ataxia syndrome (FXTAS).

## 3 Treatment recommendations

### 3.1 Treatment of movement disorders

FXTAS is a heterogeneous disorder that manifests with a variety of clinical features (3). The movement disorder manifests classically as intention tremor and cerebellar gait ataxia, with some features of parkinsonism (14). Tremor with action, posture, or rest and mixed phenotypes are seen. Parkinsonism is symmetric, with rigidity and bradykinesia as the most frequently occurring symptoms (15). Although the prevalence of parkinsonism is estimated at 29% in FXTAS patients, features like body bradykinesia (reported in 57% of cases) and postural instability (reported in 42% of cases) are quite common (16, 17). FXTAS patients may experience gait difficulties caused by a combination of cerebellar ataxia, sensory ataxia related to peripheral neuropathy, and parkinsonism. Clinical features can be measured from visit to visit with the FXTAS rating scale, which measures the salient features of the movement disorder (18).

The treatment of tremor in FXTAS is symptomatic and based on the phenotype. Owing to the phenotypical variability, several classes of medications have been tried over the years for tremor treatment in FXTAS patients. A moderate symptomatic improvement for action tremor was seen in 40% of FXTAS patients on propranolol, 50% of patients on primidone and 25% of patients on benzodiazepines (Class IV evidence) (13). Although these medications are commonly prescribed in FXTAS, side effects including sedation and worsening ataxia with the use of primidone and sedation with benzodiazpines may arise, limiting their long term use and there have been no studies looking at effectiveness when medications are combined. Clinicians may consider using essential tremor treatment algorithms or recommendations if the tremor is primarily action (19). In this situation, based on evidence in essential tremor, starting with primidone (Class I) or propranolol (Class I) could be considered depending on other patient characteristics (19). Side effects of primidone are more frequent and include sedation, nausea, ataxia, dizziness and confusion, while side effects with propranolol are less and include fatigue and bradycardia. Although topiramate can be useful in essential tremor (Class I) (19), its use is limited in FXTAS due to cognitive side effects, appetite suppression and paresthesias. Gabapentin (Class III) (19), which has been studied in essential tremor, can also be considered as the side effect profile may be favorable, but it may be less effective than primidone or propranolol. Some FXTAS patients may respond to levetiracetam (Class IV) (20) or zonisamide which has been used in essential tremor (Class III) (19), given the cerebellar features of the tremor in most patients. Rest tremor in FXTAS can be indistinguishable to that observed in Parkinson disease patients, leading to possible misdiagnoses (15). Carbidopa/Levodopa and less frequently, dopamine agonists can be used in FXTAS when rest tremor is accompanied by additional parkinsonian features, leading to modest symptomatic improvements (Class IV) (13). Botulinum toxin injections in the forearm muscles of a FXTAS patient have been reported, with modest functional and symptomatic tremor improvement (Class IV) (21), though caution should be used as botulinum toxin has not been universally effective for essential tremor and can cause weakness.

When tremor becomes refractory to oral medications, procedural interventions including deep brain stimulation (DBS, Class IV evidence in FXTAS) (22) and Magnetic Resonance Imaging guided focused ultrasound (MRIgFUS, Class IV evidence in FXTAS) (23) can be attempted, with a common target being the cerebellar relay ventral intermediate nucleus of the thalamus. However, while tremor control can be significant and sustained, undesirable side effects including worsening balance, ataxia, and cognitive impairment may arise, especially in the case of bilateral procedures, potentially limiting their use (24–26). Alternative targets, more extensive screening for cognition and ataxia at baseline, and unilateral procedures may mitigate these issues but experience and clinical trials are limited (27).

There is no universally effective treatment for cerebellar ataxia. Case reports and expert opinion suggest cerebellar ataxias may improve on amantadine (Class IV) (28). Of note, however, amantadine has anticholinergic-like side effects that require caution and careful monitoring in older patients and those with cognitive impairment. Buspirone was shown to have some benefit in a double-blind randomized controlled study for cerebellar ataxia (Class III) (29), but the advantage was not clinically significant, and subsequent studies failed to demonstrate a meaningful benefit (30, 31). In clinical practice, a trial of each could be considered with dose escalation and if not successful, they can be weaned off.

Riluzole is an N-methyl-D-aspartate (NMDA) receptor antagonist with suggested benefits for both symptoms and neuroprotection in patients with ataxia. There have been three clinical trials testing riluzole, including FXTAS patients, for treating ataxia (32–34). The combined results (Class II) indicate that riluzole may be effective for gait ataxia of various causes, but liver function should be carefully monitored during treatment (35, 36). Dalfampridine, a medication used to treat gait abnormalities in multiple sclerosis (Class I) (37) and is being investigated for other ataxias, could be considered for cerebellar ataxia if other medications are not effective (Class IV). The medication is contraindicated in patients with a history of seizures or moderate to severe renal impairment.

### 3.2 Treatment of neuropathy and other neurological signs

Up to 80% of individuals with FXTAS have an underlying length-dependent, sensory- predominant polyneuropathy (38). Patients may report numbness or neuropathic pain, and on examination they often demonstrate diminished vibratory perception in the distal lower extremities and absent Achilles reflexes (39). It is important to screen all Fragile X premutation carrier patients who present with neuropathic symptoms or signs for reversible causes of these conditions, including diabetes (through a hemoglobin A1C level or a fasting glucose tolerance test), vitamin B12 or folate deficiencies, and serum protein electrophoresis with immunofixation (40). Nerve conduction studies and peripheral skin biopsies can be helpful in evaluating pain in FXTAS patients as it helps to differentiate large fiber neuropathy and small fiber neuropathy, respectively, from other causes of pain like radiculopathy and fibromyalgia.

Management of neuropathy focuses on treating neuropathic pain and identifying and modifying additional risk factors such as hyperglycemia, paraproteinemia, and vitamin deficiencies. No specific studies have been conducted for FXTAS related neuropathy symptoms, but medications used in other neuropathic pain syndromes could be considered; gabapentin (Class I) (41), pregabalin (Class I) (42), and duloxetine (Class II) (43), or combined with amitriptyline (Class I) if monotherapy fails (44). Tricyclic antidepressants are best avoided because of the high prevalence of dysautonomia in FXTAS. Gabapentin is typically first line in other neuropathic disorders, but dosages need to be adjusted for impaired renal function and patients warned about lower extremity swelling and weight gain. Duloxetine should be used with caution in patients with renal and hepatic insufficiency. Topical analgesics such as lidocaine 5% or capsaicin 8% (Class I in other neuropathic pain disorders) (45), may provide benefit in select cases. Neuropathy contributes to gait dysfunction in FXTAS, so fall precautions should be reviewed with patients and families, emphasizing home safety and appropriate use of assistive devices such as walking sticks or walkers.

Autonomic dysfunction may occur in FXTAS and include orthostatic hypotension, constipation, erectile dysfunction, and urinary incontinence (14, 46, 47). Some patients with FXTAS have clinical syndromes that overlap with those of multiple system atrophy (48). A workup including screening of medications that might induce autonomic symptoms, assessment for reversible causes of peripheral neuropathy and cardiogenic orthostasis, and sometimes additional imaging based or sweat-based (e.g., quantitative sudomotor axon reflex) testing is indicated (49, 50).

Autonomic dysfunction in FXTAS largely aligns with the management of similar symptoms in patients with Parkinson’s disease (PD) and multiple system atrophy (MSA) (51–53). As with PD, many of these symptoms will initially respond to conservative management measures. Because no treatment trials have been conducted specifically in FXTAS, recommendations are extrapolated from PD, MSA, and general autonomic dysfunction literature. For patients with orthostatic hypotension in autonomic disorders, initial management consists of increasing hydration (Class III) and either use of compression stockings (Class III) or abdominal binders (Class III) (54). Patients should be cautioned to pause when standing to allow themselves to adjust and compensate for any orthostasis. Reduction of pro-dopaminergic, antihypertensive, and anti-diuretic medications should be strongly considered. In patients with autonomic disorders that fail these conservative approaches, use of fludrocortisone (Class III) (55) or midodrine (Class II) (56) could be considered, but patients need to be monitored for supine hypertension. Treatment for constipation, erectile and urinary dysfunction has not been tested in FXTAS, but medications used in other disorders could be considered. For constipation, many patients will respond to over-the-counter interventions or dietary changes that feature more fiber (Class I) (57), with use of prescription suppositories reserved for refractory cases. Erectile dysfunction may occur and typically responds to PDE-5 inhibitors (Class I) (58). For urinary dysfunction and incontinence, patients should first be screened for conditions such as urinary tract infections or prostate issues that might be causing or exacerbating their symptoms. Initial management strategies used in other diseases include fluid intake restrictions (Class III), timed voiding (Class II), and bladder retraining (Class II) (59). Pelvic floor therapies (e.g., Kegels, Class II) (59), typically provided by physical therapists, can be helpful. If feasible, use of oral beta-3 agonists such as mirabegron (Class I) (60) is preferred to anticholinergic agents to avoid cognitive side effects.

### 3.3 Cognitive interventions

Executive and memory dysfunction are common in FXTAS, with older age, male sex, advanced FXTAS motor stage, and medium-to-high premutation repeat size have been identified as primary risk factors for cognitive impairment, as well as more significant major neurocognitive disorder/dementia manifestations (61, 62). The memory dysfunction primarily involves recent episodic memory, particularly learning and retrieval processes (also called a dysexecutive, non-amnestic, or subcortical memory pattern). The executive dysfunction is broad, and can include specific processes such as working memory, retrieval efficiency (e.g., letter fluency), mental flexibility (e.g., inhibition), planning and sequencing, and behavioral self-regulation (61–63).

The only Class II double-blind placebo-controlled randomized clinical trial conducted in FXTAS patients tested memantine vs. placebo and the trial did not demonstrate efficacy (64). Additional pharmacological strategies have been unsuccessfully tested, such as allopregnanolone (Class IV evidence) (65), citicoline (Class IV) (66), and sulforaphane (Class IV) (67); albeit in small studies.

While cholinesterase inhibitors have been shown to improve cognition (Class I), slow decline, and delay time to negative outcomes such as death in those with dementia due to Alzheimer disease (AD) or Lewy Body disease (68, 69), only anecdotal evidence exists for treatment of FXTAS-related cognitive impairment (Class IV) (13, 70, 71). Of note, cholinesterase inhibitors have typically been recommended for those who meet criteria for major neurocognitive disorders/dementia, and *not* recommended for those with mild cognitive impairment (MCI) due to side effects overwhelming the mild-to-nil benefit (72), although more recent syntheses suggest there may be potential cognitive benefit in MCI (73). Antidepressants, particularly selective serotonin reuptake inhibitors (SSRIs), have been shown to have cognitive benefit in depressed adults, including those with late life depression (Class I) (74–76). These effects may be mediated primarily by mood improvement, and antidepressants have not demonstrated cognitive benefit in non-depressed individuals. The cognitive domains that most benefit from antidepressants include those primary affected in FXTASmemory, processing speed, and executive functioning—although the observed cognitive effects may be mediated by mood improvement (74) and cognitive benefits are not seen in non-depressed individuals (75). However, no studies evaluating the cognitive effect of antidepressants have been evaluated in FXTAS and caution may be needed given the risk of SSRIs worsening gait in some movement disorders.

Nonpharmacological cognitive rehabilitation and compensatory approaches remain a promising but underexplored therapeutic avenue for FXTAS patients with cognitive decline. In clinical practice, recommended treatments for cognitive symptoms have been synthesized into a manual by the American Congress of Rehabilitation Medicine (77). Selecting treatment is based on which cognitive domains are impacted, and to what degree the domain is impacted. Relevant to FXTAS-salient impairments, evidence-based mnemonics and particular implementations of external memory aids (e.g., planners, integrated electronic devices and envrionments) are used to ameliorate milder memory dysfunction. Approaches including metacognitive strategy training and problem-solving therapy can address milder executive dysfunction. Although no formal cognitive rehabilitation trials have been conducted specifically in FXTAS, evidence from PD (Class III) (78) and AD (Class II) (79, 80) suggest that cognitive strategy training may strengthen residual executive capacities in neurodegenerative conditions with overlapping symptomatology. Such findings support the rationale for adapting these interventions for individuals with FXTAS.

For more severe impairment, strategies used in other disorders, such as errorless learning are recommended to address memory for very circumscribed tasks, and environmental modifications and imposed structure on daily routines (e.g., task sequencing cards), can support functioning and address problematic behaviors. Additionally, Cognitive Stimulation Therapy (CST), a structured, evidence-based intervention widely used in dementia care, can support cognition, affective symptoms, and quality of life in individuals with mild to moderate (non-FXTAS) dementia (Class I) (81).

Taken together, emerging evidence and cross-condition parallels indicate that cognitive rehabilitation is an important, yet currently underutilized, component of comprehensive FXTAS care. The scarcity of randomized controlled trials and the scarcity of tailored cognitive protocols highlight the need for more systematic, integrated approaches and clearly delineate priorities for future research and clinical development.

### 3.4 Treatment of psychiatric symptoms

Neuropsychiatric features in FXTAS include apathy, anxiety, depression, disinhibition, impulsivity, irritability, mood lability, and agitation (62, 82, 83). Patients with FXTAS have significantly higher lifetime rates of mood disorders, major depressive disorder, anxiety disorders, panic disorder, social phobia, and posttraumatic stress disorder compared to the general population (83). Mood swings, irritability, and anger outbursts often occur, though bipolar disorder and psychotic signs are infrequent among people with FXTAS (84, 85). Agitation, aggression, and disinhibition can also occur in later stages of the disease, particularly in conjunction with major neurocognitive changes, warranting treatment.

#### 3.4.1 Anxiety and depression

Most therapeutic recommendations derive from clinical reports, case series, and extrapolation from general neuropsychiatric practice, given a lack of randomized controlled trials for psychiatric symptoms in FXTAS (Table 2) (86). SSRIs and SNRIs are the first-line agents in the management of generalized anxiety (Class I) and major depression (Class I) (87–89), and should be considered initially as therapeutic agents for managing these symptoms in individuals with FXTAS due to their favorable safety and tolerability profiles (90, 91). Escitalopram, sertraline, and citalopram are commonly used and preferred SSRIs, particularly because they have relatively fewer drug–drug interactions and are better tolerated in older adults (87). Fluoxetine and paroxetine are more prone to drug–drug interactions and are therefore used less frequently compared with the previously mentioned agents (92). Although SSRIs tend to have a more favorable tolerability profile compared with other classes of antidepressants, they can still produce adverse effects such as gastrointestinal discomfort, sleep disturbances, fatigue, headaches, reduced libido, exacerbate GI bleeding, QTc prolongation, worsen gait abnormalities, and increased restlessness (92–94). Specifically, clinicians should monitor for hyponatremia, sleep disturbance, extrapyramidal symptoms, and increased emotional lability, especially in older patients (92).

**Table 2:** Proposed pharmacologic treatment algorithm for anxiety and depression in FXTAS.

Step	Intervention	Key considerations
Initial assessment	Comprehensive neuropsychiatric evaluation	Confirm diagnosis, stage of FXTAS, comorbidities (e.g., anxiety, depression, sleep problems, neuropathy, autonomic dysfunction)
First-line pharmacotherapy	Start SSRI or SNRI	Choose escitalopram, sertraline, or citalopram (SSRI) or duloxetine (preferred SNRI) based on the patient’s symptom profile. Initiate at a low dose and titrate slowly.
Evaluate response and safety	After 6–12 weeks	Monitor for side effects, falls, and electrolyte disturbances
If suboptimal response or somatic anxiety	Switch within or between classes, SSRI or SNRI	Monitor blood pressure, increased bleeding risk, and irritability with SNRIs. Use venlafaxine or duloxetine; monitor blood pressure and irritability; note increased risk of bleeding with dual therapy
Persistent mood dysregulation or irritability	Behavioral strategies Consider mood stabilizer, if appropriate	Individualized decision, monitor metabolic / CNS effects
Advanced or refractory cases	Consider experimental therapies	Enrollment in clinical trials
Supportive management	Treat comorbidities	Sleep, pain, autonomic symptoms; regular monitoring; multidisciplinary team

SSRI, selective serotonin reuptake inhibitor; SNRI, serotonin–norepinephrine reuptake inhibitor.

Serotonin–norepinephrine reuptake inhibitors (SNRIs) represent a group of antidepressants that enhance the availability of the neurotransmitters serotonin and norepinephrine within the central nervous system (95). Particularly, duloxetine can be beneficial when anxiety is somatic or when depression coexists with pain (Class III, e.g., neuropathy) (43), and may be considered in individuals with FXTAS. At lower dosages, their side-effect profiles resemble that of SSRIs, commonly including gastrointestinal discomfort, fatigue or increased sleepiness, and sexual dysfunction. In contrast, when administered at higher doses, venlafaxine may lead to modest elevations in blood pressure, excessive sweating, rapid heart rate, tremors, and heightened anxiety (96). Fluoxetine, venlafaxine and duloxetine inhibit CYP2D6, so dose adjustments and close clinical monitoring are advisable when they are co-administered with medications that rely primarily on CYP2D6 metabolism (96).

Other agents, such as buspirone (Class I) (97), gabapentin (Class I) (98), mirtazapine (Class I) (99), have demonstrated benefits for reducing anxiety and various forms of psychopathological symptoms in selected populations, but their effects remain unexplored in FXTAS patients. Despite the increased risk of falls and potential for worsening cognition, benzodiazepines may be used with caution in the short-term treatment of acute anxiety or panic attacks in patients with FXTAS (Class IV) (13).

Finally, citicoline (cytidine-5’diphospho-choline) is an endogenous nucleotide which functions as a phospholipase A_2_ inhibitor and is an intermediate in the biosynthesis of structural membrane phospholipids. In an open-label trial, 10 patients with FXTAS (mean age, 70 ± 7.3 years; 90% men) were administered citicoline 1,000 mg daily for 12 months and the results showed significantly improved Beck Anxiety Inventory scores (*p* = 0.03), although the small sample size warrants further research (Class III) (66). A suggested algorithm for managing anxiety and depression in FXTAS, based on current evidence and expert consensus, is presented in Table 2.

#### 3.4.2 Obsessive compulsive disorder and symptoms

Obsessive compulsive disorder (OCD) and obsessive-compulsive symptoms (OCS) can occur in FXTAS. Obsessive compulsive symptoms are elevated in the early stages of FXTAS and rise significantly to a severity of clinical relevance by middle stage, and in men, OCS were associated with worse executive function (100). There have not been studies to evaluate treatments for OCD in FXTAS specifically, thus a general approach to treating OCD is appropriate. Fluvoxamine (Class I) and clomipramine (Class I) appear to be particularly effective in treating OCD (101), and clomipramine requires added vigilance for anti-cholinergic side effects and cardiotoxicity. Both are metabolized by CYP2D6, and about 10% of individuals have a genetic variation resulting in slower drug metabolism, which results in higher levels of drug exposure and greater risk of side effects for a given dose (102). Conversely, genetically fast metabolizers may be experiencing insufficient drug exposure even at high doses (102). In the right context, psycho-pharmacogenetic testing may be useful to inform dosing strategies.

#### 3.4.3 Psychosis

Medications for psychosis have not been studied in FXTAS. If antipsychotic medications are needed for psychosis, agitation or aggressive behavior; agents with low dopamine receptor affinity such as quetiapine, which has been studied in similar disorders (Class II) (103), may be helpful due to a lower risk of causing or worsening parkinsonism (104). Its use requires caution due to metabolic side effects, sedation, cardiovascular risk, and increased risk of death in people with major neurocognitive disorders (105, 106). Side effects may be mitigated by close risk assessment prior to prescribing, such as risk of falls in patients who may need sedating medications. Individuals with FXTAS may experience autonomic instability (61), and orthostatic hypotension is a known side effect of quetiapine, both of which could be monitored with blood pressure readings at home and review of blood pressure logs by the treating provider. A newer agent pimavanserin, a selective inverse agonist–antagonist of the serotonin 5HT2A receptor, currently approved psychosis in Parkinson’s disease (Class II) (107), may be of interest, given the lack of dopamine receptor activity (107).

SSRIs, such as sertraline (Class II) (108), citalopram (Class III) (109), or escitalopram (Class III) (109) have been studied in other disorders and can also be useful in managing irritability or agitation in people with FXTAS, particularly if anxiety or depressive symptoms co-occur. There is no evidence for the use of specific mood stabilizers for mood lability in FXTAS. Lithium has been shown to reduce behavioral symptoms in Alzheimer’s disease (Class I) (110) and could be considered, lamotrigine and valproic acid should be used with caution, given that anticonvulsants can worsen tremor and parkinsonism (111). The efficacy of anticonvulsant agents, particularly valproic acid, in managing the behavioral symptoms associated with major neurocognitive disorders/dementia has not been established (112). A recent review reported increased mortality risk among older adults with dementia in the first 3 months after initiating co-therapy with antipsychotics and valproic acid (113). As such, antipsychotic agents are recommended only when absolutely necessary and after other behavioral strategies and other pharmacological approaches have failed.

#### 3.4.4 Psychosocial and behavioral interventions

Psychosocial and behavioral interventions are a critical complement to pharmacological treatments in FXTAS management. Individuals with FXTAS with exhibit mood and anxiety symptoms, reduced stress tolerance, and progressive functional loss may respond to structured behavioral strategies (Class IV) (114). Psychotherapy provides tools that medication alone cannot offer, including psychoeducation, stress-management skills, and cognitive reframing to support adaptation to a progressive neurological condition. These interventions have not yet been systematically evaluated in FXTAS, but strong evidence from the broader psychotherapy literature, particularly for cognitive-behavioral therapy (CBT), is supportive.

CBT is a structured, time-limited intervention grounded in the assumption that maladaptive thoughts and behaviors contribute to the onset and maintenance of psychological disorders (115). By targeting and modifying these dysfunctional cognitions, CBT aims to reduce emotional distress and promote more adaptive patterns of behavior. In particular, CBT may help address catastrophic thinking, illness-related worry, and perceived loss of control, patterns frequently reported in people confronting progressive neurological decline. Preliminary support for its applicability comes from a study evaluating the FRAX-TA (Fragile X Therapeutics Assessment) program, which incorporates a single-session CBT module and was associated with significant reductions in anxiety and depressive symptoms in women with FXTAS (Class III).

Additional CBT components may be particularly relevant for FXTAS. Behavioral activation in non-FXTAS disorders can counteract apathy and inactivity (Class III), whereas exposure-based interventions (Class II), including those delivered through virtual reality, may help reduce avoidance-driven anxiety, such as fear of falling or anxiety related to medical procedures. These interventions have demonstrated efficacy in PD and may therefore represent promising approaches for individuals with FXTAS, although direct evidence is currently lacking. Importantly, given the substantial burden experienced by caregivers of individuals with neurodegenerative disorders, incorporating caregiver-focused sessions and structured support groups is also essential to foster resilience and improve outcomes for both patients and families (Class III).

Beyond traditional CBT, a range of third-wave cognitive-behavioral approaches, including Acceptance and Commitment Therapy (ACT, Class III) Mindfulness-Based Cognitive Therapy (MBCT, Class III), and Compassion-Focused Therapy (CFT, Class III), may also constitute promising avenues for psychosocial intervention in FXTAS. These modalities, which prioritize acceptance-based strategies, mindfulness practices, emotion-regulation skills, and values-guided behavioral engagement, have shown increasing utility in chronic and progressive neurodegenerative disorders. In such contexts, they assist individuals in managing illness-related uncertainty, fostering psychological resilience, and enhancing overall quality of life. Given the substantial caregiver burden associated with FXTAS, these approaches may be equally valuable for caregivers, who frequently experience sustained levels of stress, emotional exhaustion, and depressive symptomatology. When working with individuals, it is important to determine whether they have sufficient cognitive ability, as meta-analyses suggest limited benefit in those with dementia (116).

### 3.5 Rehabilitative interventions and ancillary services

#### 3.5.1 Speech and swallowing

Patients with FXTAS may be at increased risk for swallowing impairment, or dysphagia, particularly in later disease stages (116). Potential indicators of disordered swallowing include coughing, choking, food “sticking,” frequent throat clearing, and wet/hoarse vocal quality during meals, as well as prolonged mealtimes and unexplained weight loss (117). Timely intervention is imperative to avoid complications, which can include dehydration, weight loss, aspiration pneumonia, hospitalization, and mortality (118). FXTAS patients demonstrating signs/symptoms of swallowing difficulties should be promptly referred to speech-language pathology for comprehensive swallowing assessment. Bedside/clinical as well as instrumental measures, such as a modified barium swallow study and fiberoptic endoscopic evaluation of swallowing, may be employed in assessment to determine the nature and severity of swallowing difficulties and guide treatment planning. Gastroenterology may be added to the care team if esophageal pathology, such as gastroesophageal reflux disease or esophageal dysmotility, is suspected (119). Because rehabilitation trials have not been performed specifically in FXTAS, evidence grades are derived primarily from studies in degenerative cerebellar ataxias, PD, stroke, and related neurological disorders unless otherwise specified. The approach to treatment for dysphagia varies according to the underlying cause of swallowing dysfunction; therefore, evaluation by a speech-language pathologist is critical to establishing an individualized treatment plan that will yield maximal therapeutic benefit. Treatment strategies may include postural adjustments (Class II), swallowing maneuvers (Class III) (120), rehabilitative exercises (Class II) (121), dietary modifications (Class II) (122), and oral care protocols (Class II) (123). Early integration of speech-language pathology into the care team early allows for increased utilization of proactive strategies to increase functional reserve, allowing individuals to maintain swallow functioning longer in the FXTAS disease course (124).

FXTAS can also affect the voice, resulting in decreased intelligibility, effortful speech, and vocal quality that may be perceived as shaky, “quivering,” strained, “cutting out,” or having irregular pitch or volume (125). An early study of 50 men with FXTAS suggested that one in 10 participants demonstrated clinically apparent vocal tremor during neurological exam (2). Spasmodic dysphonia, a voice disorder that often co-occurs with vocal tremor, has been described in case reports of women with FXTAS (126, 127). Voice disorders result in reduced speech intelligibility and increased effort in conversation, leading to communication breakdowns, social withdrawal, and reduced quality of life (125). Men and women with FXTAS who are experiencing problems with their voice should be referred to speech-language pathology for treatment, which may include behavioral voice therapy (Class III) (128), sensory and auditory-feedback-based techniques (Class I) (129), and respiratory retraining (Class I) (130). In some cases, these strategies may be applied within a multidisciplinary management plan alongside medical treatments such as pharmacologic therapy, botulinum toxin injections, or surgical options, as appropriate (131).

Dysarthria, a problem with motor-speech coordination that results in slow, slurred, or imprecise speech, may affect over two-thirds of FXTAS patients (16). In a study of 15 men with FXTAS, the clinicians noted dysarthria in 78% during neurologic evaluation, with men with FXTAS are 6–13 times more likely than controls to score positive for speech problems on clinician rating scales. Reduced speech rate, which can be an indicator of impaired motor-speech coordination, has also been noted in both men and women with FXTAS—with no differences in severity across the sexes—and is correlated with FXTAS disease stage (132). To mitigate the impact of motor-speech deficits on communication and social functioning, FXTAS patients whose speech is difficult to understand should be referred to speech-language pathology for evaluation and treatment. Intervention may include compensatory speech strategies, augmentative and alternative communication, environmental modifications to maximize communication efficiency (all Class IV), behavioral strategies (Class III) (133, 134) to improve articulatory control and breath support and others (that have been tested in non-FXTAS patients) depending on the specific patient (135).

#### 3.5.2 Treatment for hearing loss and vestibular dysfunction

Hearing loss (including tinnitus) is reported among Fragile X premutation carriers, especially those with co-occurring depression, and may be related to FXTAS-associated disturbances in cranial nerve VIII (114, 136, 137). Case reports of FXTAS patients document unilateral and bilateral hearing loss and the presence of tinnitus (138, 139). Comprehensive hearing evaluation and intervention is critical to ensuring quality of life and enabling access to support from caregivers and clinicians. Audiologists are uniquely suited to assess, counsel, and treat hearing loss and tinnitus with device, education, and aural rehabilitation strategies. There have been no specific studies in FXTAS, but treatments may entail auditory training (Class II) (140), fitting of amplification devices for hearing loss (Class I) (141) or for tinnitus management (Class II) (142), dedicated tinnitus devices (Class III) (143), cochlear implantation and programming (Class II) (144), counseling, and psychoeducation. Such approaches may be done in collaboration with psychologists for treatment of associated depression and anxiety, speech-language pathologists for associated communication support, and ENTs and otologists for medical management.

If a patient presents with hearing loss and/or tinnitus, it is possible that vestibular involvement is also present. Audiologists, together with physical therapists, collaborate to assess possible vestibular dysfunction, as indicated by feelings of dizziness, postural instability, visual disturbance, hearing changes, tinnitus, and increased likelihood of falls. Given balance problems and gait ataxia are observed among FXTAS patients, vestibular assessment and associated treatment may provide a meaningful improvement for relevant symptoms. Importantly, vestibular functioning is linked to cognitive decline in associated conditions such as AD, so assessment and appropriate intervention is recommended to reduce the impact of potential vestibular impairment (e.g., fall risk) (145).

#### 3.5.3 Occupational therapy

Occupational therapy can help cerebellar ataxia patients, and could be considered in FXTAS, manage functional disability and maintain independence in activities of daily living, such as self-care, household management, community mobility, health management, and social participation (Class III) (146). For example, occupational therapists can provide task-specific training in daily activities such as dressing and feeding, adaptive equipment to compensate for tremor and motor problems (e.g., weighted utensils, adaptive writing tools), and strategies for managing medications and financial tasks (147). Mobility aids, environmental modifications, and assistive technologies may also be recommended to assist with fall prevention in the home, such as canes, grab bars, shower seats, or bracelets that detect falls (147, 148).

#### 3.5.4 Physical therapy

Physical therapy is critical to managing the motor symptoms of FXTAS including balance and gait instability, and difficulty with functional mobility and dual tasking (149–151). Although no studies have systematically assessed the role of physical therapy in FXTAS, a recent systematic review and meta-analysis of the degenerative cerebellar ataxias (DCA) as a whole found that physical therapy, especially muscle strengthening, coordination, balance, gait, aerobic, and activities of daily living training, may reduce DCA symptoms (Class II) (152). Gait adaptability training (with or without dual task conditions, Class IV) (153), audio-biofeedback balance training (Class IV) (154), and balance perturbation activities (Class IV) (155) may can aid in walking rhythm, postural stability, and obstacle avoidance (153, 155, 156). Two separate rehabilitation trials in DCA of various etiologies found significant improvements in balance, gait, and completion of activities of daily after a 4-week intensive rehabilitative program, with some long term carry over for months following the interventions (Class II) (157). Several small randomized controlled trials in DCA demonstrated improved gait and postural control following supervised virtual reality/exergaming protocols (Class III) (158–160). Body-weight–supported treadmill training and gait-stabilizing interventions used in cerebellar ataxias may offer additional benefit (Class IV) (161, 162).

In related neurological disorders and aging-related declines in motor function, exercise-based neurorehabilitation can improve functional decline, support quality of life, and reduce the risk of falls (Class II) (163). These outcomes may be just as clinically meaningful as improvement in the gait ataxia and if successful, may result in decreased caregiver burden. For example, a cerebellar rehabilitation trial in DCA showed improvement in functional status and ADLS and a decrease in falls, with a proportion of participants having sustained improvement out to 6 months (Class III) (164). However, a systematic review showed that therapeutic exercise may not benefit functional independence in cerebellar ataxias and more high methodological studies are needed to determine if specific ataxia exercise interventions can be successful (165). This may also be the case with the impact on quality of life, where rehabilitation interventions have been shown to improve quality of life in DCA patients, but the effects vary depending on the quality and quality of the rehabilitation (166).

Dual task cognitive and motor training paradigms (involving simultaneous performance of a motor and cognitive task) were designed with the goal of improving motor function under more ecologically valid, real-world conditions. They have been shown to improve gait, postural control, and activities of daily living, reduce falls, enhance attention and other executive functions, and improve movement automaticity and quality of life across many neurodegenerative disorders including PD, DCA, and MCI or AD (167–172). Excessive dual task costs for turning have been found in men with FXTAS (173) and a randomized controlled trial in DCA showed that integrated cognitive and balance training reduced falls and improved balance confidence in DCA (Class III) (170). A dual task cognitive-motor high intensity body-weight–supported treadmill training study was initiated in FXTAS patients in 2019, but had to be discontinued due to the Covid-19 pandemic (Class IV) (174).

Physical therapists may also provide recommendations on the use of assistive devices, such as canes and walkers, orthotics, and elastic garments or weight vests (Class IV) (175) to reduced ataxia and improve stability. These devices may improve functional mobility, independence, and safety across environmental contexts in other disorders. Periodic assessment and reassessment of a need for assistive devices is important as disease progression may change needs over time. The choice of assistive device should take into account not only the severity of ataxia, but also balance, upper-limb function, the presence of neuropathy, cognitive status, fall risk, and the environmental context in which the individual performs daily activities. In this context, as the disease progresses, rehabilitation goals may gradually shift from functional recovery or improvement toward compensation, safety, preservation of independence, and reduction of caregiver burden. Emerging adjunctive therapies, such as dry needling, acupuncture, or other physical therapy-based modalities may have utility and can be considered, but need to be investigated for effectiveness inFXTAS patients in the future.

Emerging and investigational interventions such as non-invasive brain stimulation techniques, including transcranial direct current stimulation (tDCS) and repetitive transcranial magnetic stimulation (rTMS), may represent promising adjunctive rehabilitative interventions for FXTAS, although direct evidence is currently lacking. In DCA and other neurodegenerative conditions, cerebellar and motor cortex stimulation protocols have shown potential benefits on gait, balance, motor coordination, mood symptoms, and cognitive functioning (Class III) (176). Proposed mechanisms include modulation of cerebello-thalamo-cortical networks, enhancement of neuroplasticity, and facilitation of motor learning. Preliminary studies in mild cognitive impairment and depression also suggest potential cognitive and affective benefits of non-invasive neuromodulation (Class II) (177). However, evidence remains preliminary, stimulation protocols are heterogeneous, and safety and efficacy specifically in FXTAS require systematic investigation.

## 4 Nutrition and exercise

No lifestyle intervention trials have been conducted specifically in FXTAS and the following recommendations are extrapolated from studies in DCAs and related neurodegenerative disorders. From a nutritional standpoint, deficiencies in folate and vitamin B12 have been linked to cognitive decline, and elevated homocysteine levels to increased neuronal vulnerability to oxidative stress (178). Vitamin levels in the blood can be checked and B-complex supplementation given if levels are low. The Mediterranean-Dietary Approach to Systolic Hypertension (MIND) diet was created as a tailored diet to protect the brain. This diet has now been extensively studied and is associated with slower cognitive decline with age (Class III) (179). The MIND diet components include 10 brain healthy food groups (green leafy vegetables, other vegetables, nuts, berries, beans, whole grains, seafood, poultry, olive oil and wine) and five unhealthy food groups (red meats, butter and stick margarine, cheese, pastries and sweets, and fried/fast food) (179). This diet may be most pertinent to recommend to FXTAS patients for brain protection and to lower hypertension, but studies to show a direct impact on cognitive function are needed.

Exercise remains one of the most promising non-pharmacological interventions for FXTAS, supported by extensive evidence from other DCA and neurodegenerative diseases (Class II) (180). A recent RCT in persons with DCA demonstrated that home-based high-intensity aerobic cycling training improved ataxia symptoms and aerobic fitness and reduced fatigue significantly more than dose equivalent balance training (Class II) (181). Importantly, benefits were maintained in the aerobic group if they trained regularly for 1 year. A similar study showed that 30 min of cycling exercise at 65–85% of maximum heart rate at least 4x/week increased step length compared to traditional balance training (Class II) (182). A summary of interventions is also provided in Table 3.

**Table 3:** Cognitive and behavioral interventions for FXTAS.

Domain	Intervention	Description	Clinical considerations in FXTAS
Cognitive	Cognitive rehabilitation	Structured cognitive exercises targeting attention, working memory, task sequencing, and problem solving.	Best for mild–moderate cognitive impairment; may require caregiver involvement; sessions should be shorter due to cognitive fatigue.
	Compensatory strategies	External memory aids, calendars, visual cueing systems, step-by-step task lists, environmental restructuring.	Enhances independence; reduces frustration; essential as disease progresses.
	Assistive technology	Cursor stabilization, adaptive keyboards, voice-to-text, weighted utensils.	Mitigates tremor-related functional impairment; improves performance in computer tasks.
Psychotherapy	Traditional cognitive behavioral therapy (CBT)	Targeted treatment for anxiety, depression, maladaptive beliefs, avoidance, and behavioral withdrawal.	Highly effective; must be adapted for slowed cognition, using visual supports and shorter sessions.
	Acceptance and commitment therapy (ACT)	Focuses on psychological flexibility, values-based action, mindfulness.	Well suited for progressive neurological decline; reduces distress even without cognitive restructuring.
	Mindfulness-based interventions	MBCT, mindfulness training, stress-reduction practices.	Reduces stress reactivity; may indirectly reduce tremor exacerbation; helpful for caregivers.
	Caregiver psychotherapy	CBT/ACT/MBCT for caregiver stress, burnout, and depressive symptoms.	Reduces caregiver distress and improves patient outcomes indirectly.

MBCT, mindfulness-based cognitive therapy.

## 5 Limitations

Overall, there is a paucity of FXTAS-specific treatment studies or randomized trials with a high level of evidence, lack of standardized outcome measures and limited evidence for long-term treatment effects, all of which creates an opportunity for researchers in this area. However, this creates a reliance on extrapolating treatment recommendations from other disorders that can be problematic as it is not specific. Heterogeneity in the disorder is also problematic, including differences in affected men and women. Other limitations include that the writing team was created from attendees at the International Fragile X Premutation meeting, with addition of specific expertise that was needed as the paper was written. This compellation of experts may have excluded other clinicians or researchers who work in this area and were either not in attendance at the meeting or not known/invited to the author team. Finally, knowledge regarding the change of FXTAS over time is lacking. Two ongoing longitudinal studies will address this by evaluating both men and women with FXTAS and their clinical trajectories over a 5 year time period and this will allow clinicians a better understanding of how treatment needs may also change as the disease progresses.

## 6 Conclusion

Given the multisystemic nature of FXTAS, a comprehensive, multidisciplinary treatment model is essential for effective management of FXTAS patients. Optimal care requires coordinated contributions from neurologists, psychiatrists, psychotherapists, neuropsychologists, speech-language pathologists, physical, and occupational therapists, and other rehabilitation specialists to address the full spectrum of neurological, psychiatric, physical, and psychosocial needs.
